# Development and validation of a novel analytical method for related substances of resmetirom and identification of new degradation products

**DOI:** 10.3389/fchem.2026.1795082

**Published:** 2026-03-24

**Authors:** Jisu Qin, Qunfeng Luo, Wenyi Wu, Qin Wang, Liangliang Cai, Shuang Wang, Yonghong Zhu

**Affiliations:** 1 Department of Pharmacy, Affiliated Nantong Hospital of Shanghai University (The Sixth People’s Hospital of Nantong), Nantong, China; 2 School of Pharmacy, Nantong University, Nantong, China; 3 School of Basic Medical Sciences, Jiangxi Medical College, Nanchang University, Nanchang, Jiangxi, China; 4 Department of Quality Inspection, Sinopharm Holding Nantong Ltd., Nantong, China; 5 Department of Pharmacy, Affiliated Hospital of Nantong University, Nantong, China

**Keywords:** method development, method validation, novel products, related substances, resmetirom

## Abstract

**Background:**

Resmetirom (MGL-3196), a highly selective thyroid hormone receptor β agonist, is indicated for treating adults with noncirrhotic nonalcoholic steatohepatitis (NASH) exhibiting moderate to advanced liver fibrosis. Related substances are critical factors that directly impact the safety and efficacy of the active pharmaceutical ingredient (API). Therefore, the quantitative detection of related substances in resmetirom is of particular importance.

**Methods:**

This study established a reverse-phase high-performance liquid chromatography (RP-HPLC) for the separation of resmetirom and its related substances. The analysis was performed using an Agilent 5 HC C_18_ (2) column (250 mm × 4.6 mm, 5 µm) at 35 °C, with detection at 220 nm. A gradient elution method was employed, achieving effective separation of all impurities within a 45-min runtime. The final HPLC method utilized with a mixture of H_2_O/ACN (90/10, v/v) containing 0.045% TFA as mobile phase A, and ACN as mobile phase B. The gradient elution proceeded as follows: 0–2 min, 10% B→ 10% B; 2–20 min, 10% B→ 50% B; 20–30 min, 50% B→ 54% B; 30–35 min, 54% B→ 54% B; 35–36 min, 54% B→ 10% B; and 36–45 min, 10% B→ 10% B. The rate of flow was 1.0 mL/min. A 10 µL volume was used for the sample injection.

**Results:**

The method was validated according to International Conference on Harmonization (ICH) guidelines, and the results demonstrated that it possesses good specificity, stability, linearity, precision, repeatability, and robustness. Furthermore, a new degradation product, designated as imp-A, was generated under alkaline degradation conditions in this study. Its chemical structure was elucidated using nuclear magnetic resonance (NMR) and high-resolution mass spectrometry (HRMS), and this compound has been reported for the first time.

**Conclusion:**

The RP-HPLC method established in this study is capable of accurately and reliably separating and detecting resmetirom and its related substances. The method is simple to operate and yields dependable results, providing a practical and efficient analytical tool for the quality control of resmetirom and its formulations. This contributes to ensuring the safety and efficacy of the drug product.

## 1 Introduction

Noncirrhotic nonalcoholic steatohepatitis (NASH), also known as metabolic dysfunction-associated steatohepatitis (MASH) (Targher et al., 2021), is a progressive liver disorder characterized by hepatic steatosis, inflammation, hepatocellular injury, and escalating fibrosis (Chalasani et al., 2018; Loomba et al., 2021; Rinella et al., 2023). The risk of adverse clinical outcomes rises significantly when NASH progresses to advanced fibrosis, especially in individuals with type 2 diabetes (Angulo et al., 2015; Dulai et al., 2017; Huang et al., 2023; Targher et al., 2021). The prevalence of NASH is closely linked to the growing obesity epidemic (Konerman et al., 2018; Younossi et al., 2018). While lifestyle modifications can help manage related metabolic disorders, they are often difficult to implement and maintain (Chalasani et al., 2018; Vilar-Gomez et al., 2015).

In this context, the thyroid hormone receptor β (THR-β) plays an important role, particularly in liver cells. THR-β is crucial for regulating metabolic processes that are often disrupted in NASH (Sinha et al., 2019). Research suggests that reduced thyroid hormone levels in the liver may contribute to NASH, with both clinical and subclinical hypothyroidism being more common in NASH patients compared to age-matched controls (Bohinc et al., 2014; Sinha et al., 2019).

This background led to the approval of resmetirom by the U.S. Food and Drug Administration on 14 March 2024, as a treatment for NASH in adults with moderate to severe liver fibrosis (stages F2 to F3) (FDA, 2024). Resmetirom (MGL-3196) is an orally taken drug that selectively activates THR-β, with about 28 times more preference for THR-β than THR-α (Kelly et al., 2014). It was developed from the pyridazinone series, which showed higher THR-β selectivity than earlier compounds. The introduction of a cyanoazauracil group further enhanced its effectiveness and selectivity (Kelly et al., 2014). In NASH, selectively activating THR-β may provide metabolic benefits mainly through liver actions, reducing potential negative effects on the heart and bones (Sinha et al., 2019). Clinical studies suggest that improvements in fibrosis in patients treated with resmetirom likely stem from reduced metabolic damage rather than direct antifibrotic effects (Wirth et al., 2022). Additionally, resmetirom has a favorable safety profile with low toxicity in preclinical studies and generally does not affect thyroid function, making it well-tolerated by patients (Dutta et al., 2024; Harrison et al., 2019).

However, to our knowledge, no straightforward methods for the analysis of resmetirom-related substances have yet been reported. The synthetical compound and degradation of resmetirom can lead to impurities that are significant for both safety and therapeutic efficacy, necessitating thorough consideration in process preparation studies and quality control. To enhance the detection of resmetirom-related substances, the development of a robust method is imperative.

Reversed-phase high-performance liquid chromatography (RP-HPLC), a powerful method for drug and impurity identification, is widely used in the pharmaceutical industry (Annadi et al., 2022; Hardeep et al., 2022; Reçber et al., 2022; Zhao et al., 2022). Therefore, this study developed and validated a method using RP-HPLC for the determination of resmetirom. The method is simple to operate, highly sensitive, accurate, and robust, enabling effective separation of related substances of resmetirom, including process-related impurities and degradation products (designated as imp-A, imp-B, imp-C, and imp-D). Furthermore, evaluations of specificity, precision, and robustness were carried out, and the limit of detection, limit of quantification, linear range, and recovery rates of the method were established. Notably, this study identified impurity A as a previously undocumented degradation product, potentially representing a novel compound. This research provides an effective analytical method for quality control of resmetirom active pharmaceutical ingredient (API).

## 2 Materials and methods

### 2.1 Materials, chemicals and reagents

Resmetirom along with imp-B, -C, -D were obtained from Shanghai Caerulum Pharma Discovery Co., Ltd, while imp-A was prepared in the authors’ lab. Anaqua Chemicals Supply provided Acetonitrile (ACN) and methanol (MeOH) of HPLC grade. Trifluoroacetic acid (TFA) (analytical reagent) was from Shanghai Aladdin Biochemical Technology Co., Ltd. Hydrochloric acid (HCl), phosphoric acid, sodium hydroxide (NaOH), hydrogen peroxide (H_2_O_2_), and triethylamine (analytical grade) were sourced from China National Pharmaceutical Group Corporation.

### 2.2 Instruments

RP-HPLC separation and analysis were performed with an Agilent 1,200 instrument from Agilent in the United States, which was fitted with a UV–vis spectrometer (Varian Cary 300, United States). Furthermore, the study utilized a Milli-Q water purification system.

### 2.3 Chromatographic conditions

Resmetirom and its related compounds were separated using an Agilent 5 HC C_18_ (2) column (250 mm × 4.6 mm, 5 µm), operating at a temperature of 35 °C. The final HPLC method utilized a gradient elution with a mixture of H_2_O/ACN (90/10, v/v) containing 0.045% TFA as mobile phase A, and ACN as mobile phase B. The gradient elution proceeded as follows: 0–2 min, 10% B→ 10% B; 2–20 min, 10% B→ 50% B; 20–30 min, 50% B→ 54% B; 30–35 min, 54% B→ 54% B; 35–36 min, 54% B→ 10% B; and 36–45 min, 10% B→ 10% B. The wavelength for UV detection was set at 220 nm. The rate of flow was 1.0 mL/min. A 10 µL volume was used for the sample injection.

### 2.4 Preparation, purification and structural confirmation of imp-A

#### 2.4.1 Preparation of crude imp-A

To obtain imp-A, 100 mg of resmetirom was precisely weighed and placed in a 250 mL round-bottom flask. 100 mL of methanol was added and stirred until complete dissolution. Subsequently, 50 mL of 1 M sodium hydroxide solution was added, and the mixture was allowed to react at room temperature for 1 h. After the reaction, hydrochloric acid was slowly added dropwise under stirring to adjust the mixture pH to approximately 7.0. Methanol was removed by rotary evaporation. The remaining aqueous solution was subjected to freeze-drying, yielding a salt-containing crude product. This crude product was dissolved in water to prepare a 300 mg/mL solution, stirred to ensure complete dissolution, and then centrifuged at 3,000 rpm for 20 min. The precipitate was separated and further freeze-dried to obtain the crude imp-A.

#### 2.4.2 Purification of imp-A

An appropriate amount of the above crude product was dissolved in a 75% (v/v) methanol aqueous solution via sonication to prepare a 15 mg/mL solution. Purification was performed using an Agilent 1,260 preparative HPLC system equipped with an automatic fraction collector. The chromatographic column was an Agilent Eclipse XDB-C18 (250 mm × 9.4 mm, 5 μm). The mobile phase was 60% (v/v) acetonitrile aqueous solution at a flow rate of 3 mL/min. Detection was performed at a wavelength of 220 nm over a run time of 25 min. Fractions eluting between 9.5 and 10.7 min were collected. The collected fraction solutions were combined, concentrated by rotary evaporation to remove acetonitrile, and then freeze-dried to obtain purified imp-A. The purity of Impurity A was determined according to the method described in Section 2.3.

#### 2.4.3 Structural characterization of imp-A

The structure of imp-A was elucidated using nuclear magnetic resonance (NMR) spectroscopy and high-resolution mass spectrometry (HRMS). For NMR analysis, 10–20 mg of imp-A was dissolved in deuterated dimethyl sulfoxide (DMSO-d6). 1H NMR, 13C NMR, and 2D NMR spectra were recorded on a Bruker Avance NEO 600 MHz spectrometer (Bruker Corporation, Billerica, MA, United States) equipped with a broadband observe probe at 298 K. HRMS analysis was conducted using a ThermoScientific Q Exactive instrument. The sample was injected via a sample loop (2 µL), with methanol as the mobile phase at a flow rate of 200 μL/min. Full-scan high-resolution mass spectra were acquired in both positive and negative ion modes using the Q Exactive HESI ion source. The mass resolution was set to 140,000 full width at half maximum at m/z 200. The spray voltage was set to 3.50 kV (positive ion)/3.00 kV (negative ion), with sheath gas and auxiliary gas flow rates of 45 and 15 arbitrary units, respectively. The transfer capillary temperature was maintained at 250 °C, and the S-Lens RF level was set to 50 V. Data acquisition and processing were performed using Xcalibur software.

### 2.5 Preparation of stock solution

#### 2.5.1 Standard stock solution of resmetirom

An exact weight of 25 mg of resmetirom was dissolved in a mixture of H_2_O and MeOH (25/75, v/v). The mixed liquid was then moved to a 100 mL container and diluted to the mark to obtain a standard solution with a concentration of 0.25 mg/mL.

#### 2.5.2 Standard stock solutions of resmetirom-related impurities

Resmetirom impurities A, B, C, and D were accurately weighed and then diluted with 75% aqueous methanol solution to a concentration of 0.25 mg/mL to prepare the standard stock solution.

### 2.6 Preparation of mixed solution and system suitability solution

To create a combination solution with resmetirom bulk drug and its related substances, around 25 mg of the bulk drug was weighed and put into a 100 mL volumetric flask. Next, 1 mL of each reference standard stock solution of impurities imp-A, B, C, and D was added. The solution was diluted to 100 mL using a mixture of H_2_O and MeOH (25/75, v/v).

The system suitability solution was prepared by oxidative degradation products and stock solutions of imp-B. Oxidation degradation products were generated by introducing 15% H_2_O_2_ into the resmetirom solution at a temperature of 55 °C. After 5 h, the oxidative decomposition product was achieved, followed by the addition of a suitable quantity of imp-B.

### 2.7 Preparation of sample solution

About 25 mg of resmetirom was precisely measured and mixed with a solution of water and methanol in a 25:75 ratio to create a sample solution with a concentration of 0.25 mg/mL.

## 3 Results

### 3.1 Method development

**FIGURE 1:**
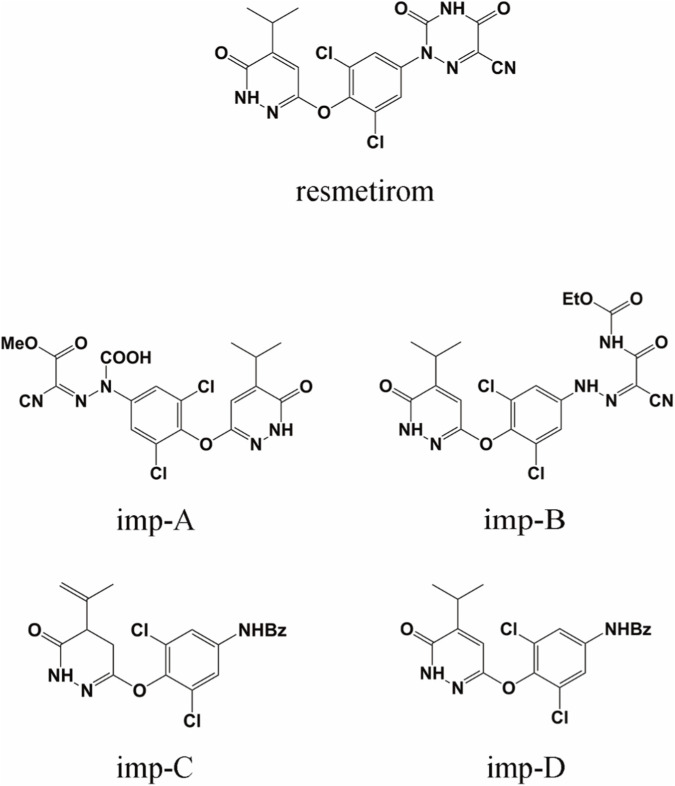
Chemical structures of resmetirom and its known process-related and degradation impurities.

The synthetic scheme for resmetirom is presented in Supplementary Figure S1. The synthesis involves various raw materials and intermediates. Four major related substances were identified through forced degradation studies: imp-A, imp-B, imp-C, and imp-D. The chemical structures of resmetirom and imp A-D are depicted in Figure 1. Intermediate products 5, 6, and 8 (identified as imp-C, imp-D, and imp-B, respectively) were categorized both as process-related impurities and as degradation products. Imp-A was characterized as a base-induced degradation product. A method using RP-HPLC was created and confirmed for identifying compounds associated with resmetirom in this study.

#### 3.1.1 Choice of solvent

Dissolution experiments reveal that resmetirom is insoluble in water, exhibits slightly soluble in acetonitrile, and is sparingly soluble in methanol. Consequently, the solubility of resmetirom in methanol is superior to that in acetonitrile. Furthermore, resmetirom (1 mg/mL) in methanol remains stable without significant changes observed at 12, 24, 48, or 72 h. Given the potential environmental impact of methanol, the study explored various concentrations of methanol-water solutions as solvents. It was observed that a 50% methanol-water solution induced precipitation of the active pharmaceutical ingredient, resmetirom, whereas a 75% methanol-water solution improved its solubility. Therefore, considering the solubility, stability, and cost-effectiveness of resmetirom in different solvents, a 75% methanol-water mixture was selected as the optimal solvent.

#### 3.1.2 Wavelength selection

**FIGURE 2:**
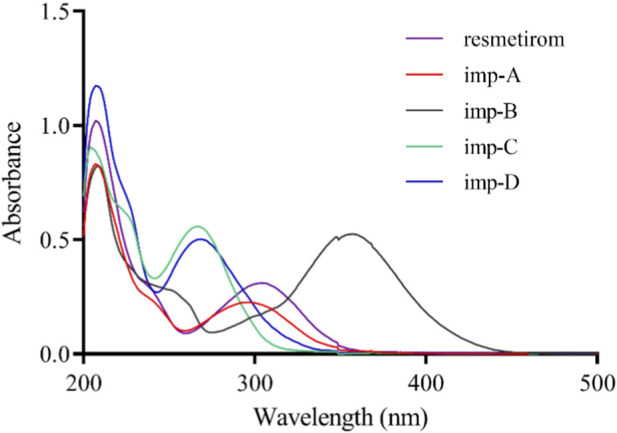
Ultraviolet (UV) absorption spectra of resmetirom and its known process-related and degradation impurities.

To determine the optimal absorption wavelength, UV-visible spectra of resmetirom and its impurities (10 μg/mL) were obtained from 200 nm to 500 nm. The UV spectra were recorded, as illustrated in Figure 2. All five substances exhibited significant absorption near 220 nm. Consequently, 220 nm was chosen as the detection wavelength.

#### 3.1.3 Chromatography column selection

Different C_18_ columns of lengths 150 mm and 250 mm were tested in preliminary experiments with mobile phase systems consisting of H_2_O and ACN. The key information related to the HPLC columns that were used in screening studies is summarized in Table 1. As anticipated, the chromatographic resolution for critical pairs was improved when using a 250 mm long column compared to a 150 mm long column. Examining chromatograms from various C_18_ columns (250 mm × 4.6 mm, 5 µm) shows that the Agilent 5 HC C_18_ (2) column (250 mm × 4.6 mm, 5 µm) produced more peaks with improved resolution and lower back pressure in H_2_O-ACN mobile phase setups.

**TABLE 1:** List of HPLC columns used for screening in the HPLC method development of resmetirom and its impurities.

Column	Manufacturer	Dimension (mm)	Particle size (μm)	Pore size (Å)
5HC- C_18_ (2)	Agilent	150 × 4.6	5	170
Spherigel- C_18_	Morhchem	150 × 4.6	5	80
SinoChrom ODS-BP- C_18_	Elite	150 × 4.6	5	120
Luna-C_18_ (2)	Phenomenex	250 × 4.6	5	100
Gemini-NX-C_18_	Phenomenex	250 × 4.6	5	110
XBridge-C_18_	Waters	250 × 4.6	5	110
5HC-C_18_ (2)	Agilent	250 × 4.6	5	170

#### 3.1.4 Adjustment of elution method

Based on a preliminary structural analysis indicating that resmetirom is acidic in nature, a trace amount of trifluoroacetic acid (TFA) was added to the mobile phase to suppress analyte ionization and to mask residual silanol groups on the stationary phase, thereby improving separation efficiency and peak symmetry. Solubility studies demonstrated that resmetirom is more soluble in methanol than in acetonitrile, which would theoretically favor methanol as the organic modifier. However, owing to the relatively low polarity of the compound, the use of methanol resulted in prolonged retention times. In contrast, acetonitrile provides stronger elution strength in reversed-phase chromatography. Under the low-loading conditions employed (sample concentration 0.25 mg/mL, injection volume 10 μL), the analyte remained fully soluble in the initial mobile phase. Considering these factors, acetonitrile was ultimately selected as the organic component of the mobile phase. After multiple rounds of optimization and verification, mobile phase A was defined as a mixture of 0.045% (v/v) TFA in water and acetonitrile (9:1, v/v), while acetonitrile was used as mobile phase B.

**FIGURE 3:**
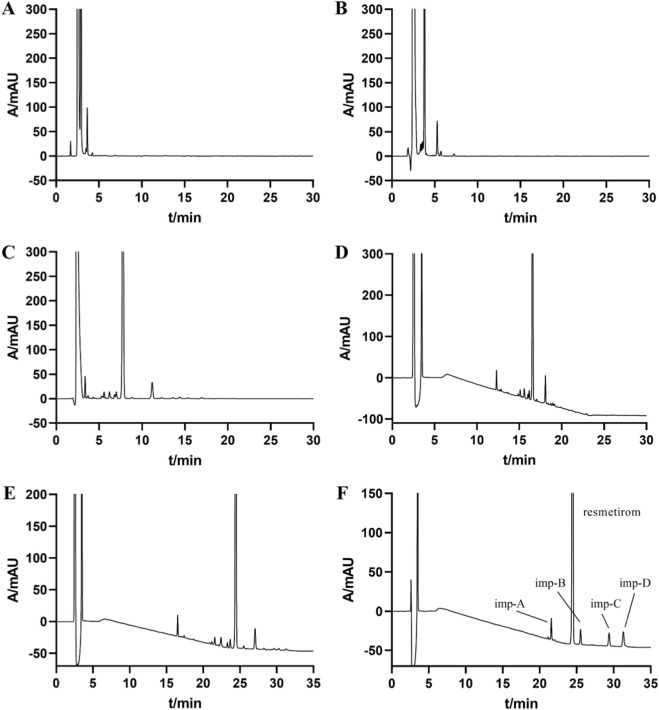
HPLC chromatograms obtained during the optimization of chromatographic conditions for the determination of resmetirom and its related impurities: **(A)** Chromatogram obtained under isocratic elution condition 1; **(B)** Chromatogram obtained under isocratic elution condition 2; **(C)** Chromatogram obtained under isocratic elution condition 3; **(D)** Chromatogram obtained under gradient elution condition 1; **(E)** Chromatogram obtained under the final optimized gradient elution condition; **(F)** Chromatogram of resmetirom and its known related impurities (imp-A, imp-B, imp-C, imp-D) acquired under the final gradient elution condition.

Because the system suitability solution contained numerous impurities at relatively high concentrations, including several critical peak pairs that were difficult to resolve, subsequent optimization of the elution program was primarily performed using this solution. To simplify operation and maintain baseline stability, isocratic elution was first investigated, as it eliminates column re-equilibration and enhances analytical efficiency and reproducibility. Three isocratic conditions were evaluated, with mobile phase A/mobile phase B ratios of 10:90 (isocratic elution condition 1), 30:70 (isocratic elution condition 2), and 50:50 (isocratic elution condition 3). As shown in Figures 3A–C, increasing the proportion of the aqueous phase improved the resolution between resmetirom and its related substances and increased the number of detectable impurity peaks. Nevertheless, complete separation of all components could not be achieved under isocratic conditions.

Accordingly, various gradient elution programs were further explored. Application of Gradient Condition 1 (0–2 min, 10% B; 2–20 min, 10%–90% B; 20–30 min, 90% B; 30–31 min, 90%–10% B; 31–40 min, 10% B) resulted in a minimum resolution of 0.97 between adjacent impurity peaks, which failed to meet the acceptance criterion of not less than 1.2 (Figure 3D). The gradient program was therefore systematically optimized, ultimately establishing the chromatographic conditions described in Section 2.3. Under the optimized conditions, the resolution between the principal peak and all impurity peaks satisfied the predefined criteria, as illustrated in Figure 3E.

### 3.2 Method validation

The developed HPLC method for analyzing resmetirom was validated according to the International Conference on Harmonization (ICH), encompassing specificity, linearity and range, accuracy, precision, sensitivity, and robustness.

#### 3.2.1 Specificity

The specificity of the method is essential for the successful development of an RP-HPLC technique. Accordingly, an analysis of the mixed solution and system suitability solution was conducted to evaluate the method’s specificity as the conditions in Section 2.3.

A chromatogram of the system suitability solution is illustrated in Figure 3E. The resolution between the main peak and nearby impurity peak were 3.75 and 4.88, respectively, both exceeding 1.5. The minimum resolution between impurity peaks exceeded 1.2, measuring at 1.84.

The chromatogram for the solution containing mixed impurities is depicted in Figure 3F, where peaks 1–5 correspond to imp-A, resmetirom, imp-B, imp-C, and imp-D, respectively. The resolutions between the main peak, resmetirom, and its nearest impurities were 14.69 and 4.88, both exceeding the threshold of 1.5. Among the impurity peaks, the lowest resolution recorded was 5.81, surpassing the minimum acceptable value of 1.2. Therefore, these findings indicate that the suggested approach is suitable for the system.

#### 3.2.2 Forced degradation tests

In the forced degradation tests, approximately 25 mg of resmetirom was accurately weighed and transferred to a 100 mL volumetric flask, then dissolved in H_2_O/MeOH (25/75, v/v). The sample underwent various stress factors including acid and base hydrolysis, oxidation, photolytic, and thermal stress. Specific conditions included acid hydrolysis using 2 mol/L HCl for 7 days at ambient temperature; base hydrolysis with 0.1 mol/L NaOH for 0.3 h at room temperature; oxidative stress with 15% H_2_O_2_ for 5 h at 55 °C; thermal stress at 100 °C for 7 days; and photolytic stress using an LED tube at 4,500 lux for 30 days. Post-treatment, the samples were diluted to 100 mL with H_2_O/MeOH (25/75, v/v) and analyzed as per the protocols in Section 2.3.

**FIGURE 4:**
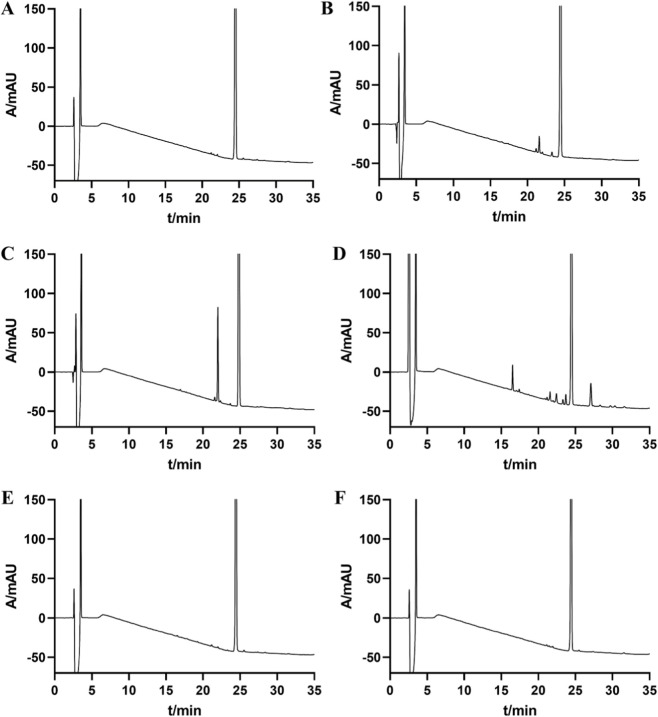
HPLC chromatograms of resmetirom under forced degradation conditions: **(A)** non-degraded; **(B)** acid-degraded; **(C)** base-degraded; **(D)** oxidatively degraded; **(E)** heat-degraded; **(F)** photolytically degraded.

Figure 4 displays the outcomes of the forced degradation test using chromatography. Resmetirom’s stability was assessed under various forced degradation conditions based on the number of impurities, peak content, the minimum resolution between main peak and impurities, and the minimum resolution among impurities (Supplementary Table S1). Resmetirom was relatively stable under acid, heat and light stress but was easily degraded under basic and oxidation conditions. The main peak demonstrates a minimum resolution of greater than 1.5 compared to adjacent impurity peaks, and the resolution between impurity peaks exceeds 1.2, which satisfies the criteria. The equilibrium ranges from 95.8% to 103.6%. Typically, the material balance rate falls within the 95%–105% range, indicating a state of basically equilibrium.

#### 3.2.3 Limit of detection and quantification

The LOD represents the minimal level of analyte that can be qualitatively detected, while the LOQ is the minimal concentration that can be quantitatively measured with specified levels of recovery and precision. In this study, resmetirom standard stock solution, along with reference standards for related substances, were serially diluted and injected to determine the signal-to-noise (S/N) ratio. After six injections, the concentrations that corresponded to S/N ratios of 3:1 and 10:1 were determined as the LOD and LOQ, respectively. The summarized results are presented in Table 2.

**TABLE 2:** Linearity, LOD and LOQ of resmetirom and its known impurities (imp-A, imp-B, imp-C, imp-D).

Substance	Standard calibration curves	Correlation	LOD	LOQ	Linearity range
		Coefficient (r)	(μg/mL)	(μg/mL)	(μg/mL)
Resmetirom	y = 44.051x + 2.3027	0.9999	0.02	0.05	0.05–5.09
Imp-A	y = 39.05x - 0.2386	0.9998	0.02	0.05	0.05–5.07
Imp-B	y = 26.272x + 0.0606	0.9999	0.03	0.09	0.09–5.18
Imp-C	y = 41.776x-0.544	0.9999	0.02	0.07	0.07–5.11
Imp-D	y = 56.755x + 15.713	0.9999	0.01	0.03	0.03–5.05

#### 3.2.4 Linearity and range

This study investigated the linearity of resmetirom and its related substances across a concentration range from the quantitative limit to 2.0% of the intended analytical concentration (∼0.25 mg/mL). Sample solutions containing impurities A, B, C, and D were prepared in a mixture of H_2_O/MeOH (25/75, v/v) at series concentrations and subjected to analysis. The LOD, LOQ, linear ranges, and regression equations for resmetirom and its related substances are presented in Table 2. The correlation coefficients (r) of all calibration curves exceeded 0.99, indicating good linearity. These results demonstrate that the analytical method shows no significant deviation in the quantification of resmetirom and its related substances within the concentration range from the quantitative limit to 2.0%.

#### 3.2.5 Precision

To assess precision, mixed solutions prepared for a stability study were analyzed six times, recording RT and peak areas for resmetirom and imp A-D. Resmetirom and its related substances (imp-A, -B, -C, -D) had RSDs of 0.07%, 0.08%, 0.08%, 0.10%, and 0.11% for their RT values. The RSDs for the peak areas are 0.66%, 1.23%, 1.27%, 1.94%, and 1.71% in that order. Every RSD is below 2%, showcasing the accuracy of the device.

#### 3.2.6 Repeatability

In order to evaluate the repeatability of the RP-HPLC technique, a combination solution was created in six replicates and analyzed under the specified conditions outlined in Section 2.3. The RSDs for the components of resmetirom, imp-A, -B, -C, and -D are 1.05%, 1.33%, 1.41%, 1.30%, and 1.47%, all falling below 2%, demonstrating the method’s strong repeatability.

#### 3.2.7 Recovery

The recovery of resmetirom-related substances was evaluated at three concentration levels: 50%, 100%, and 150%. Resmetirom sample solutions at different levels were spiked with related substance solutions, with each concentration being prepared in triplicate. Following this, the samples were injected and the percentage of recovery was determined. Table 3 presents the recovery results for each related substance. The recovery rates of all substances were within the range of 95%–110%. These results indicate a high level of method accuracy.

**TABLE 3:** Recovery results of resmetirom known impurities (imp-A, imp-B, imp-C, imp-D).

Target Level	Spiked Conc. (μg/mL)	Determined Conc. (μg/mL)	Recovery (%)	Average recovery	RSD (%)
50%	0.253	0.245	96.84	100.88	2.79
	0.253	0.246	97.23		
	0.253	0.249	98.42		
100%	0.506	0.523	103.16		
	0.506	0.531	104.73		
	0.506	0.512	100.99		
150%	0.759	0.781	102.76		
	0.759	0.769	101.18		
	0.759	0.772	101.58		
50%	0.259	0.261	100.77	99.04	2.27
	0.259	0.252	97.30		
	0.259	0.268	103.47		
100%	0.518	0.496	95.75		
	0.518	0.509	98.26		
	0.518	0.505	97.49		
150%	0.777	0.775	99.87		
	0.777	0.769	99.10		
	0.777	0.774	99.74		
50%	0.256	0.265	103.52	102.34	1.78
	0.256	0.259	101.17		
	0.256	0.264	103.13		
100%	0.512	0.526	102.94		
	0.512	0.539	105.48		
	0.512	0.531	103.91		
150%	0.768	0.771	100.52		
	0.768	0.764	99.61		
	0.768	0.781	101.83		
50%	0.252	0.245	97.22	98.91	2.61
	0.252	0.251	99.60		
	0.252	0.251	99.60		
100%	0.504	0.509	100.79		
	0.504	0.507	100.40		
	0.504	0.495	98.02		
150%	0.756	0.761	100.53		
	0.756	0.702	92.73		
	0.756	0.759	100.26		

#### 3.2.8 Robustness

Considering the potential for minor variations in chromatographic parameters during the method’s lifecycle, it is crucial to evaluate their impact on critical chromatographic attributes. To this end, the robustness of the chromatographic conditions was assessed by deliberately introducing slight modifications to the HPLC parameters. System suitability tests were conducted to verify the method’s resilience under varied chromatographic scenarios. Detailed specifications of these conditions are provided in Supplementary Table S2.

Modest adjustments to the chromatographic conditions yielded a minimum resolution of 2.44 (>1.5) between resmetirom and its adjacent impurity peaks, confirming adequate separation. For other impurity peaks, besides the significant effects of changing the chromatographic column, the minimal separation degree under other conditions was 1.26 (>1.2), indicating satisfactory separation. No significant changes were observed in the impurity profiles. Variations in chromatographic parameters such as the mobile phase composition, detection wavelength, column temperature, flow rate, and column type showed minimal impact on the detection of related substances. These results demonstrate the method’s robustness, as minor chromatographic modifications did not substantially affect the resolution and RT of resmetirom and associated substances.

### 3.3 Characterization of imp-A

**FIGURE 5:**
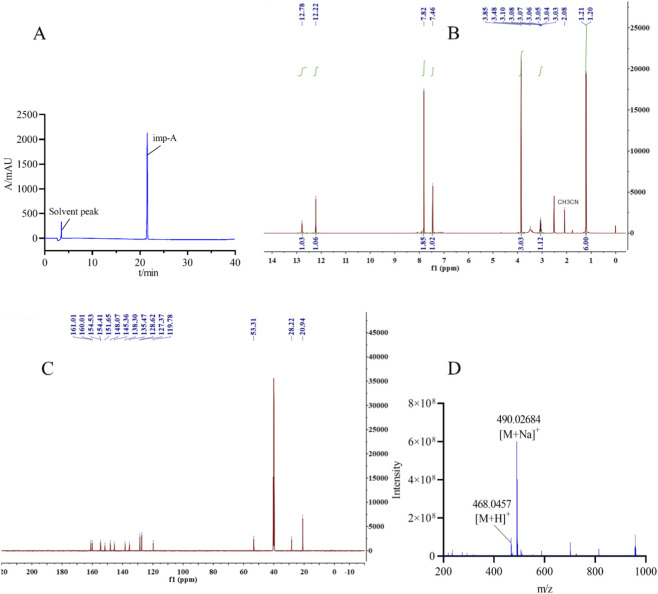
Structural characterization of imp-A: **(A)** HPLC chromatogram; **(B)** ^1^H NMR spectrum; **(C)** ^13^C NMR spectrum; **(D)** HRMS spectrum.

Imp-A was prepared in the authors’ laboratory as described in Section 2.4. Its chromatographic profile is shown in Figure 5A, exhibiting a retention time of 21.58 min and an estimated purity of 99.1%. Imp-A was structurally characterized by NMR and HRMS analyses, which enabled the identification and quantification of its chemical composition. The ^1^H NMR and ^13^C NMR spectra are presented in Figures 5B,C, respectively, and the HRMS spectrum is shown in Figure 5D. The ^1^H and ^13^C NMR signal assignments of imp-A are summarized in Table 4. In addition, the 2D NMR spectrum is provided in Supplementary Figure S2. Based on the combined HRMS and NMR data, the molecular structure of imp-A was elucidated, as shown in Figure 1.

**TABLE 4:** ^1^H and^13^C NMR assignments of imp-A.

δ (ppm)	Number of H atom	Structure
1.21	H1-2	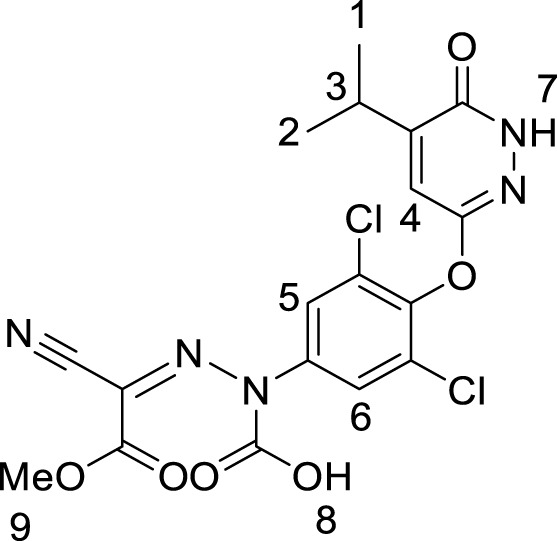
2.97–3.17	H3	
3.85	H9	
7.82, 7.46	H4-6	
12.22	H7	
12.78	H8	
​	​	
20.94	C1,C2	
28.22	C3	
53.31	C9	
161.01, 160.01, 154.53	The other C atoms	
154.41, 151.65, 148.07		
145.36, 138.30, 135.47		
128.62, 127.37, 119.78		

## 4 Conclusion

This study focused on creating a dependable and efficient RP-HPLC technique to identify resmetirom (MGL-3196), a medication recently approved for treating NASH, along with any impurities present. Validation of this detection method, following the ICH guidelines, demonstrated excellent specificity, high sensitivity, as well as satisfactory linearity, precision, repeatability, and robustness. Additionally, a novel degradation product was discovered, and its structure was successfully elucidated. The aim for this research to provide valuable insights for future studies on resmetirom, the initial drug approved for treating NASH.

## Data Availability

The original contributions presented in the study are included in the article/Supplementary Material, further inquiries can be directed to the corresponding authors.
