# Supplementary material for: Development and validation of a novel analytical method for related substances of resmetirom and identification of new degradation products
Source: Front Chem. 2026 Mar 24;14:1795082. doi: 10.3389/fchem.2026.1795082 (PMC13053519; doi:10.3389/fchem.2026.1795082)
Supplement: Supplementary file 1 [file DataSheet1.docx]

Supplementary Material


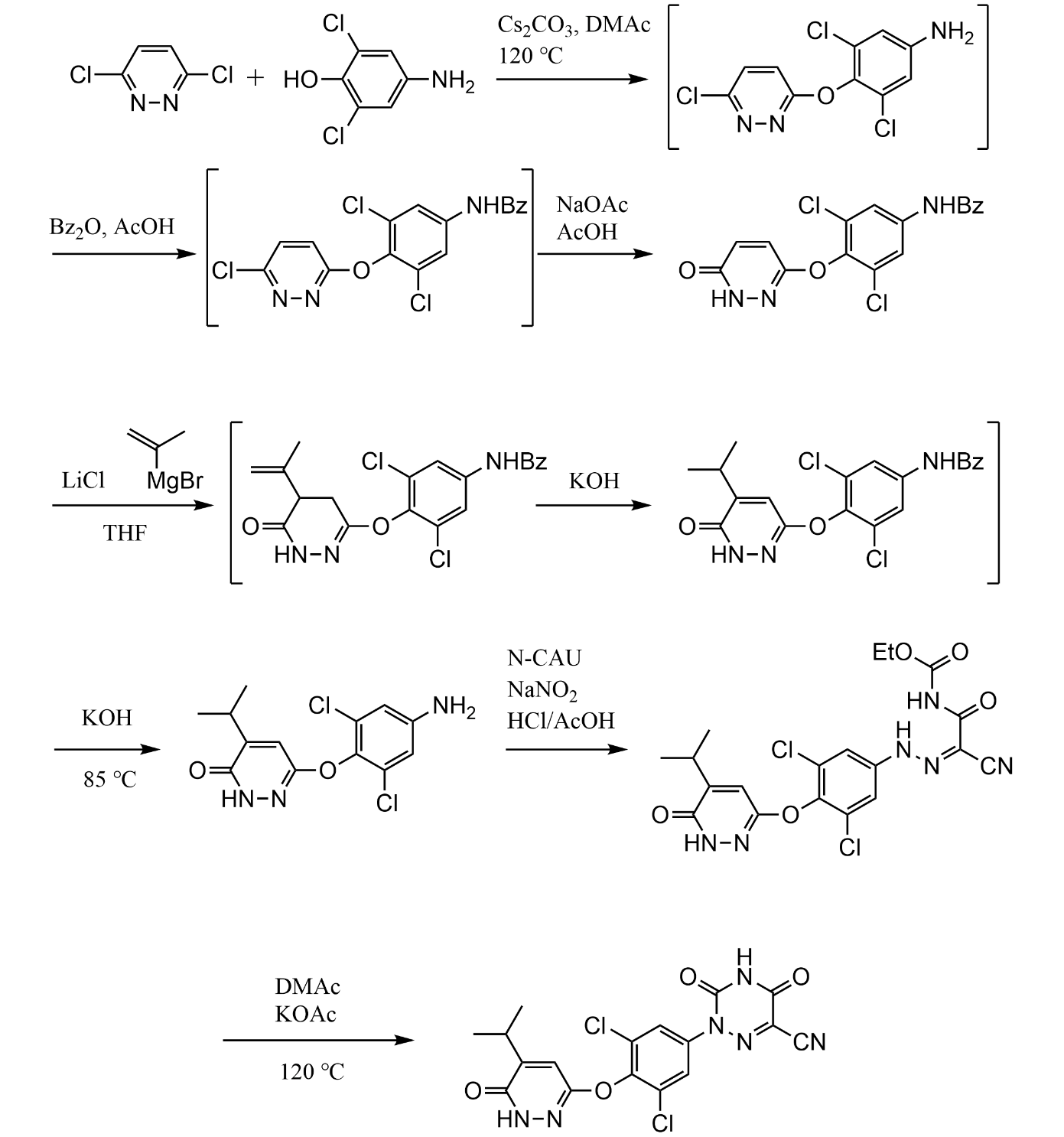


**Figure S1.** The synthetic scheme for resmetirom


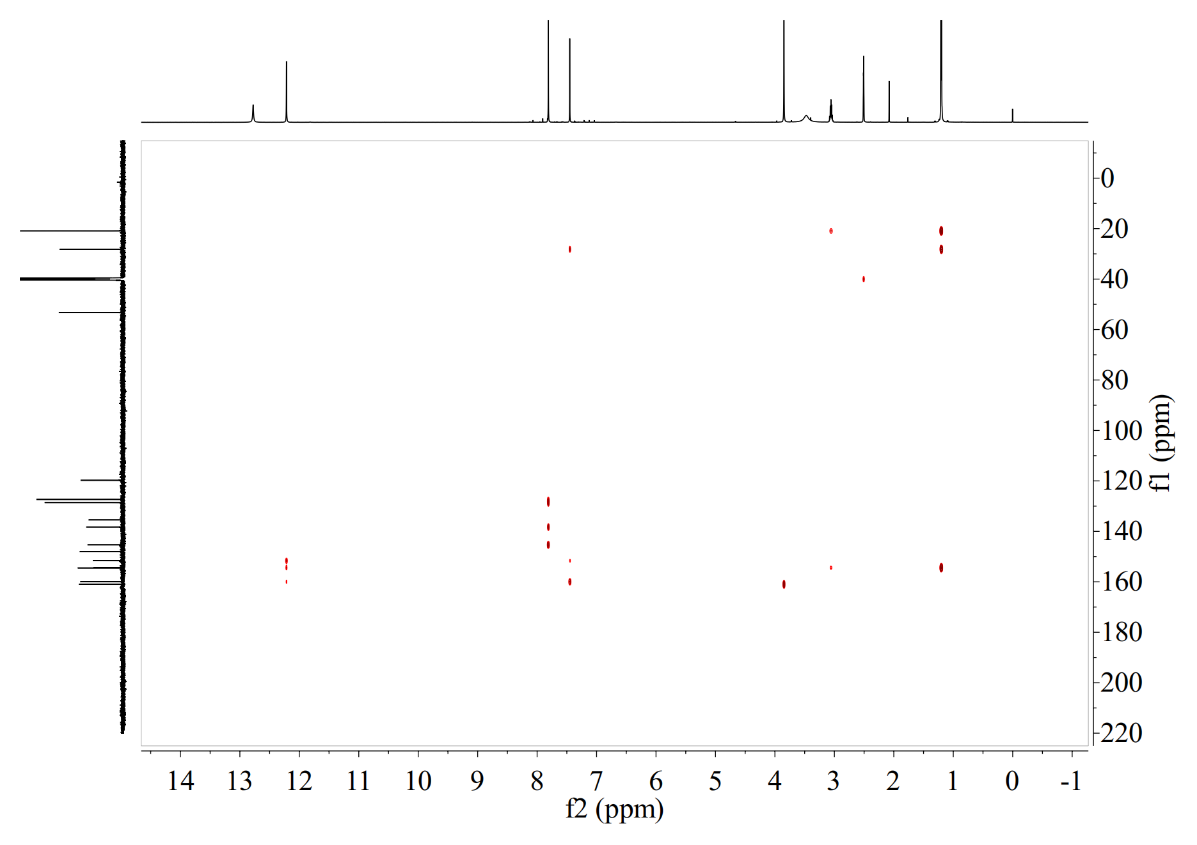


**Figure S2.** 2D NMR spectrum of imp-A

**Table S1** Results of forced degradation tests for resmetirom

| Forced Degradation Condition | Number of impurities (>0.03 %) | Content of main peak (%) | Minimum resolution between main peak and impurities | Minimum resolution among impurities | Equilibrium (%) |
| --- | --- | --- | --- | --- | --- |
| Undegradation | 6 | 99.26 | 4.94 | 1.77 | 100 |
| Acid degradation | 7 | 97.84 | 5.46 | 2.32 | 99.1 |
| Base degradation | 6 | 91.44 | 5.91 | 1.98 | 97.3 |
| Oxidative degradation | 14 | 89.08 | 3.78 | 1.86 | 95.8 |
| Heat degradation | 7 | 98.99 | 4.84 | 4.63 | 103.6 |
| Photolytic degradation | 6 | 99.25 | 4.90 | 1.80 | 98.8 |

**Table S2** Test conditions for robustness.

| **Chromatogram conditions** | **The variation range of parameters** |
| --- | --- |
| The initial proportion of mobile phases A-B (%) | 92: 8, 90: 10, 88: 12 |
| Wavelength (nm) | 215, 220, 225 |
| Column temperature (℃) | 30, 35, 40 |
| Flow rate (mL/min) | 0.9, 1.0, 1.1 |
| Chromatographic column | Gemini-NX-C_18_, Agilent-5HC-C_18_, XBridge-C_18_ |
